# Lack of association between mitochondrial DNA polymorphisms and didexoxynucleoside-induced hyperlactataemia in black-African, HIV-1-infected patients

**DOI:** 10.1186/1758-2652-13-S4-P96

**Published:** 2010-11-08

**Authors:** A Arenas-Pinto, I Weller, R Ekong, A Grant, A Karstaedt, L Telisinghe, M Bolhaar, S Charalambous, N Bradman, C Ingram

**Affiliations:** 1 University College London, Centre for Sexual Health & HIV Research, London, UK; 2 University College London, Centre for Genetic Anthropology, London, UK; 3 London School of Hygiene & Tropical Medicine, Clinical Research Unit, London, UK; 4 Chris Hani Baragwanath Hospital, Johannesburg, South Africa; 5 The Aurum Institute, Johannesburg, South Africa

## Background

Recent studies have shown some association between specific mitochondrial DNA (mtDNA) polymorphisms and peripheral neuropathy in both white European and black American populations. An association between mtDNA haplogroup H and peripheral lipoatrophy has been reported in white Europeans. Our group has shown a lack of association between mtDNA polymorphisms and the occurrence of HL in white Europeans exposed to dideoxynucleosides, but there have been no studies in black African people.

## Methods

mtDNA was extracted from oral mucosa epithelial cells from HIV1-infected active patients seen at Chris Hani Baragwanath Hospital in Johannesburg, South Africa. Cases were defined as confirmed blood lactate >5 mmol/l. Controls were randomly selected from patients who had never developed HL. Sequencing of the hypervariable region 1 (HVS-1) of the mtDNA was performed on all samples and the haplotypes obtained were reported as differences from the Cambridge Reference Sequence. Specific single nucleotide polymorphisms (SNP) were used to predict haplogroup. Logistic regression was used to identify variables associated with case status. An exact test of population differentiation was used to compare HVS-1 haplotype distribution between cases and controls.

## Results

mtDNA was obtained from 40 cases and 38 controls. 82.5% of cases and 63.2% of the controls were female (P= 0.05). The ethnicity of the majority of participants were self-defined as Zulu or Sotho (80% of cases and 63% of controls; P=0.184) and all of them were exposed to either d4T (100% of cases and 87% of controls) or AZT (13% of controls). The median blood lactate level in cases at the time of diagnosis was 7.65 mmol/l (IQR 6.65-9.45). The distribution of mtDNA haplotypes was not different between cases and controls (P=0.137) neither were the predicted haplogroups (P=0.429).

After adjusting for haplogroup distribution and ethnicity only factors known to be associated with HL, such as female gender (OR 5.92; 95%CI 1.50-23.42) and CD4 count <200 cell/ml (OR 4.02; 95%CI 1.03-15.68) remained independently associated with case status.

## Conclusions

We did not find any association between mtDNA polymorphisms and the occurrence of HL in black African adults exposed to dideoxynucleosides. Contrary to what has been published on other mitochondrial toxicities, our data suggest that mtDNA variability may not explain any predisposition for dideoxynucleoside-associated HL.

